# *GSTM1 *and *APE1 *genotypes affect arsenic-induced oxidative stress: a repeated measures study

**DOI:** 10.1186/1476-069X-6-39

**Published:** 2007-12-04

**Authors:** Carrie V Breton, Molly L Kile, Paul J Catalano, Elaine Hoffman, Quazi Quamruzzaman, Mahmuder Rahman, Golam Mahiuddin, David C Christiani

**Affiliations:** 1 Harvard School of Public Health, Dept of Environmental Health, 665 Huntington Avenue, Boston, MA 02115, USA; 2 University of Southern California, Keck School of Medicine, 1540 Alcazar Street, CHP 236 Los Angeles, CA 90033, USA; 3 Dana-Farber Cancer Institute, 375 Longwood Avenue 2nd floor, Boston, MA 02115, USA; 4 Harvard School of Public Health, Dept of Biostatistics, 655 Huntington Avenue, Boston, MA 02115, USA; 5 Dhaka Community Hospital, 190/1 Baro Moghbazar, Wireless Railgate, 1217, Dhaka, Bangladesh

## Abstract

**Background:**

Chronic arsenic exposure is associated with an increased risk of skin, bladder and lung cancers. Generation of oxidative stress may contribute to arsenic carcinogenesis.

**Methods:**

To investigate the association between arsenic exposure and oxidative stress, urinary 8-hydroxy-2'-deoxyguanosine (8-OHdG) was evaluated in a cohort of 97 women recruited from an arsenic-endemic region of Bangladesh in 2003. Arsenic exposure was measured in urine, toenails, and drinking water. Drinking water and urine samples were collected on three consecutive days. Susceptibility to oxidative stress was evaluated by genotyping relevant polymorphisms in glutathione-s transferase mu (*GSTM1*), human 8-oxoguanine glycosylase (*hOGG1*) and apurinic/apyrimidinic endonuclease (*APE1*) genes using the Taqman method. Data were analyzed using random effects Tobit regression to account for repeated measures and 8-OHdG values below the detection limit.

**Results:**

A consistent negative effect for *APE1 *was observed across water, toenail and urinary arsenic models. *APE1 148 glu/glu + asp/glu *genotype was associated with a decrease in logged 8-OHdG of 0.40 (95%CI -0.73, -0.07) compared to *APE1 148 asp/asp*. An association between total urinary arsenic and 8-OHdG was observed among women with the *GSTM1 *null genotype but not in women with *GSTM1 *positive. Among women with *GSTM1 *null, a comparison of the second, third, and fourth quartiles of total urinary arsenic to the first quartile resulted in a 0.84 increase (95% CI 0.27, 1.42), a 0.98 increase (95% CI 033, 1.66) and a 0.85 increase (95% CI 0.27, 1.44) in logged 8-OHdG, respectively. No effects between 8-OHdG and toenail arsenic or drinking water arsenic were observed.

**Conclusion:**

These results suggest the *APE1 *variant genotype decreases repair of 8-OHdG and that arsenic exposure is associated with oxidative stress in women who lack a functional *GSTM1 *detoxification enzyme.

## Introduction

Arsenic, a naturally occurring element, is a common environmental contaminant. Elevated concentrations can occur in drinking water, the primary route of exposure, through natural and anthropogenic process. Many countries are affected by arsenic-contaminated groundwater including Argentina, Australia, Chile, China, Hungary, India, Mexico, and the United States. However, the most affected region is Bangladesh, where an estimated 29 to 40 million people are at risk of ingesting arsenic-contaminated drinking water [1]. While epidemiological studies have clearly demonstrated arsenic as a human carcinogen, the mechanisms of toxicity remain largely unknown [2].

One hypothesized mechanism involves the induction of oxidative stress through the generation of reactive oxygen species (ROS) [3]. Animal and *in vitro *experiments have shown that arsenic induces oxidative stress in the liver, brain and blood of rats, increases oxidant levels in cultured aortic endothelial cells, increases intracellular peroxide levels, and reduces antioxidant levels [4-6]. Furthermore, mice that have been administered the arsenic metabolite dimethylarsinic acid (DMA V) orally and topically have developed skin tumors and shown increased levels of 8-hydroxyguanine in the epidermis [7].

Whether chronic arsenic exposure in humans causes oxidative stress is less certain. In a small cross-sectional study of Chinese community residents exposed to drinking water arsenic, oxidative stress levels, measured by mean serum levels of lipid peroxide, were significantly higher among the arsenic-exposed group [8]. Non-protein sulfhydrl groups such as glutathione in serum, which act as nucleophilic scavengers and protect against oxidative damage, were also inversely correlated with mean serum arsenic levels. In another study, blood arsenic levels correlated positively with the level of plasma oxidant species and inversely with plasma antioxidant capacity [9]. Several epidemiologic studies have also demonstrated an association between arsenic and the oxidative biomarker, 8-hydroxy-2'-deoxyguanosine (8-OHdG) [10,11]. In addition, skin tissue studies have shown significant differences in 8-OHdG concentration when comparing arsenic exposed and unexposed skin samples [12].

8-OHdG is a byproduct of ROS damage to DNA which can cause mutation of G:C to T:A if it remains in the DNA at the time of replication. 8-OHdG in urine is a widely accepted marker of oxidative DNA damage and oxidative stress [13]. Normal DNA repair removes 8-OHdG adducts which are excreted and measurable in urine, blood and tissues. The urinary levels of oxidized products of nucleic acid breakdown thus reflect the amount of DNA damage incurred by ROS and repaired by the body's normal repair processes [14].

Numerous genes influence the generation and repair of oxidative lesions. Some of the most well-studied include genes in the base excision repair (BER) pathway and glutathione-s transferase (GSTs) [15]. Polymorphisms in these genes may affect the association between arsenic and 8-OHdG. Specifically, 8-OHdG lesions are repaired by human 8-oxoguanine glycosylase (*hOGG1*) in combination with apurinic/apyrimidinic endonuclease (*APE1*) [16]. Variants in the two genes *hOGG1 Ser326Cys *and the *APE1 Asp148Glu *have demonstrated a decreased ability to repair oxidative damage [17]. *GSTs*, a superfamily of multifunctional enzymes involved in cellular detoxification, conjugate and eliminate electrophilic carcinogens and scavenge free radicals [18]. A role for glutathione-s transferase *mu (GSTM1*) in DNA repair has also been suggested [19]. A homozygous deletion in the *GSTM1 *gene results in lack of enzyme and is generally hypothesized to increase the accumulation of cellular DNA damage [20]. *GSTs *have also been associated with an increased risk for oxidative stress-related diseases. For example, *GSTM1 *null genotype was associated with cutaneous basal cell carcinoma and with solar keratoses [19,21].

Studies have demonstrated that the magnitude of disease risk is greater when the *GSTM1 *null genotype interacts with other factors [20]. Because homozygous deletions in *GSTM1 *have been associated with changes in arsenic methylation capability [22], *GSTM1 *genotype may alter arsenic toxicity and therefore modify the association between arsenic and urinary 8-OHdG.

In the present study, we investigated whether chronic arsenic exposure was associated with oxidative stress by evaluating the association between total urinary arsenic (TUA), toenail arsenic and drinking water arsenic and urinary 8-OHdG concentration among a cohort of Bangladeshi women who did not exhibit any cutaneous signs of arsenic toxicity and were exposed to a wide range of arsenic concentrations in drinking water. In addition, we evaluated effect modification of several genotypes including *hOGG1 Ser326Cys, APE1 Asp148Glu*, and *GSTM1 *null.

## Methods

### Participant Selection

Participants were recruited through a series of community meetings held in three villages in Pabna, Bangladesh that are serviced by the Pabna Community Clinic. Overall, 248 individuals from 50 households were recruited to participate in a repeated measures study to characterize biomarker response. Details regarding the full cohort are described elsewhere [23]. Briefly, subjects were eligible for this study if they were long-term residents of Pabna, obtained their drinking water from tube wells, and received primary health care from the Pabna Community Clinic, an affiliate of Dhaka Community Hospital (DCH). During the initial visit, a behavioral and demographic questionnaire was administered and a blood sample collected. Researchers visited participants at their homes every three months to collect urine, toenail, and water samples.

For this study, the cohort was restricted to women who provided urine samples from April 15, 2003 to June 27, 2003. The cohort was restricted to women because they spent more time at home and thus may have a more consistent exposure profile. These restrictions yielded 97 women and 290 urine samples for analysis. All women provided three consecutive days of urine samples except one, who only provided two samples.

Institutional Review Boards at the Harvard School of Public Health and DCH approved the protocol for this study. Informed consent was obtained prior to participation.

### Arsenic measurement

First void urine samples were collected from participants and stored at -20°C until analysis. Multiple collections were used to reduce the effect of day-to-day variations in background levels of 8-OHdG. TUA was measured using high performance liquid chromatography-atomic absorption spectrophotometry (HPLC-HGAAS; HPLC model Waters 501, Waters Associates, Milford, MA, USA) as described by Hsueh *et al *[24]. This method quantifies arsenate (As V), arsenite (As III), monomethylarsonic acid (MMA), and dimethylarsenic acid (DMA). TUA was defined as the sum of As III, As V, MMA, and DMA. Detection limits for As III, As V, MMA, and DMA were 0.036, 0.055, 0.054, and 0.056 μg/L, respectively.

Drinking water concentrations from family tube wells were measured on each day of urine collection. Water samples were collected in acid-washed polyethylene containers and preserved with Reagent Grade HNO_3 _(Merck, Germany) to a pH < 2. Total inorganic arsenic analysis was performed by Environmental Laboratory Services (North Syracuse, New York) following U.S. Environmental Protection Agency method 200.8. The limit of detection for this method is 1 μg As/l. Measurements that fell below the limit of detection were assigned a value of half the limit of detection for statistical analyses. PlasmaCAL multi-element QC standard #1 solution (SCP Science, Canada) was used to validate analysis.

Toenail clippings were collected from all toes and prepared as described by Chen *et al *[25]. Arsenic was analyzed using an inductively-coupled plasma mass spectrometer (ICP-MS Model 6100 DRC, Perkin-Elmer, Norwalk, CT). Standard reference material water (NIST 1643d Trace Elements in Water; National Institute of Standards and Technology, Gaithersburg, MD) and certified human hair reference material (CRM Hair; Shanghai Institute of Nuclear Research, Academia Sinica, China) were used to validate instrument performance and digestion method. The average percent recovery of NIST 1643 and CRM hair was 92.4% and 94.5%, respectively.

The reported inorganic arsenic concentrations were corrected for any detectable blank concentrations and for systemic error by normalizing the sample concentrations against the measured average daily NIST 1643 inorganic arsenic concentration [23]. This corrected value was used in all the statistical analyses.

### 8-OHdG measurement

One milliliter of urine was shipped overnight on dry ice to Genox Laboratories (Baltimore, MD) for analysis. 8-OHdG was measured using an ELISA assay, a competitive immunoassay in which color intensity is inversely proportional to the concentration of 8-OHdG in the urine samples [26]. A pooled urine sample from several healthy adults was used as the in-house quality control. Triplicate measurements were performed on all samples, their values averaged, and any sample with a percent coefficient of variation (SD/mean expressed as a percentage; %CV) equal or greater than 20% was re-tested. The limit of detection for this method was 0.64 μg/l. Several methods are available for measurement of 8-OHdG, including ELISA, HPLC-ECD, LC/MS/MS and GC/MS. Although some evidence suggests the ELISA may be less sensitive and overestimate values compared to other methods, it is a readily available and easy assay which has demonstrated good correlation between 8-OHdG values measured by both HPLC-ECD and LC/MS/MS [27].

### Creatinine measurement

Creatinine was measured using the Sigma Diagnostics creatinine assay (Genox Laboratories) from the same sample used for 8-OHdG analysis. This assay measures creatinine by a kinetic modification of the Jaffe reaction [28].

### Genotyping

Multiplex polymerase chain reaction amplifications were performed from genomic DNA extracted from whole blood following the Puregene Protocol (Gentra Systems, Minnesota). Genotyping of the *GSTM1*deletion followed the protocol described by Liu *et al *[29]. The following primers were used: 5'-CGCCATCTTGTGCTACATTGCCCG-3' and 5'-TCTGGATTGTAGCAGATCA-3'. The *hOGG1 Ser326Cys *(rs1052133) and *APE1 Asp148Glu *(rs3136820) polymorphisms were detected by the Taqman method using the ABI Prism 7900HT Sequence Detection System (Applied Biosystems, Foster City, CA). Primers for *hOGG1 *and *APE1 *were as follows: a) 5'-ACAGACTCCACCCTCCTACAG-3', b) 5'-ACCCTTTCTGCGCTTTGCT-3', c) 5'-GATTGCTTTCCCTTTTCTTATAGTTTTTTATGCT-3', and d) 5'-CACCCGGCCTTCCTGAT-3', respectively. Genotyping procedures were validated by randomly selecting 5% of the samples and subjecting them to repeat analysis. Two researchers independently reviewed all genotyping results and samples with unclear results were rerun until 100% concordance was achieved.

### Statistical analysis

All analyses were conducted using Tobit regression (STATA, version 9.2, College Station, TX). This method was chosen because of its ability to validly account for left-censored data since 25% of 8-OHdG values in our dataset were below the limit of detection [30]. Models included a random effect to account for the correlation between repeated urine measures. Arsenic, 8-OHdG and creatinine concentrations were strongly right skewed but appeared to be lognormal when transformed using a natural logarithm. TUA, toenail arsenic and drinking water arsenic concentrations were initially treated as continuous variables in all models, then categorized into quartiles to evaluate potential non-linear associations. Estimated betas and their corresponding 95% confidence intervals were computed for the parameters of interest according to the following model:

ln (8-OHdG_ij_) = *α*_*ij *_+ *β*_*As *_ln As_ij _+ *β*_*G *_Gene_ij _+ *β*_*Z *_Z_ij _+ b_j_^(2) ^+ e_ij_,

where α_*ij *_is a baseline intercept for the *i*^th ^observation in the *j*^th ^woman; As_ij _is arsenic in drinking water, toenails, or urine; Gene_ij _is a main effect for *GSTM1, APE1 *or *hOGG1 *genotype; Z_ij _is a vector of covariates; b_j_^(2) ^is a random person effect; and the β coefficients are the parameters to be estimated.

All models were adjusted for potential confounding by age, BMI, education, creatinine, betel nut chewing and the presence of at least one smoker in the household. Chewing tobacco, drinking tea, childhood tan level and childhood skin reaction to approximately two hours of sun exposure were initially evaluated for potential confounding but then dropped from subsequent models because none of these variables appreciably changed the effect estimates for arsenic. None of the women reported smoking cigarettes themselves so confounding by smoking was not relevant.

Lastly, effect modification of the association between arsenic and 8-OHdG by polymorphisms in *GSTM1, APE1*, and *hOGG1 *was evaluated by including interaction terms in the model above. Heterozygous and homozygous variant genotypes were combined and compared to their corresponding common variants for all genes. Stratum-specific effect estimates and confidence intervals were calculated directly from models that included interaction terms. For the models in which arsenic was categorized into quartiles, Wald p-values were presented for specific quartile-gene interactions and likelihood ratio tests were performed to test whether the group of arsenic variables and the group of gene-arsenic interactions as a whole were associated with 8-OHdG levels.

## Results

Sociodemographic information for the 97 Bangladeshi women who provided 290 urine samples included in this analysis are provided in Table 1. The majority of women were married (82.5%) and had only a primary education level or lower (71.2%). None of the women reported smoking, although 51.6% reported having at least one smoker in the household and 28.9% reported chewing betel nuts. Prevalence of the variant genotypes was 4.2% for *APE1 *glu/glu, 13.8% for *hOGG1 *cys/cys, and 27.8% for *GSTM1 *null. The median value of TUA was 22.6 (μg/l), though a wide range (1.35 – 431.3 μg/l) was observed (Table 2). When urinary 8-OHdG samples were analyzed, 25% were below the limit of detection. The median 8-OHdG value was 3.25 μg/l (range 0.64 – 82.6 μg/l). 8-OHdG measurements from each woman exhibited a fair degree of variability. The average %CV was 55.1% with a range from 4.0 to 152.8%. The adjusted within-person correlation for daily measurements, calculated directly from the Tobit model in Table 3, was 0.47.

**Table 1:** Sociodemographic and genetic characteristics of 97 women from Pabna, Bangladesh

	**%**	**N**
Marital status		
Married	82.5	80
Married	8.3	8
Widowed	9.3	9
Education		
Illiterate	15.5	15
Able to write	39.2	38
Primary	16.5	16
Secondary	17.5	17
Higher 2nd	9.3	9
College/graduate	2.1	2
Smokers in the household	51.6	50
Chew betel nuts	28.9	28
*hOGG1 Ser326Cys*		
Cys/cys	13.8	13
Cys/ser	44.7	42
Ser/ser	41.5	39
*APE1 Asp148Glu*		
Glu/glu	4.2	4
Asp/glu	32.6	31
Asp/asp	63.2	60
*GSTM1*		
Null	27.8	27
Positive	72.2	70

**Table 2:** Exposure distributions and physical characteristics of 97 women from Pabna, Bangladesh

	**mean**	**SD**	**median**	**range**
***urinary measures***				
Total urinary arsenic (μg/l)^†^	39.5	53.1	22.6	1.35 – 431.3
8-OHdG (μg/l)*^†^	6.1	9.0	3.3	0.64 – 82.6
Creatinine (mg/dl)^†^	48.3	33.9	39.4	4.3 – 230.5
***toenail measure***				
Toenail arsenic (μg/g)	1.4	1.5	0.8	0.22 – 10.16
***drinking water measure***				
Water arsenic (μg/l)	64.7	115.9	1.46	0.5 – 591
***physical characteristics***				
Age	34.8	12.9	33.0	17 – 70
BMI	21.3	3.6	21.4	12.9 -30.3

*25% of 8-OHdG values were below the LOD.

^†^repeated measurements for each woman were included separately for a total of 290 values

**Table 3:** Main effects* of arsenic and covariates on logged urinary 8-OHdG (μg/l) (N = 290)

	**Urine**		**Drinking water**		**Toenail**	
**Variable**	**β**	**95% CI**	**β**	**95% CI**	**β**	**95% CI**

Arsenic quartile						
1st	reference		reference		reference	
2nd	0.16	(-0.15, 0.46)	0.02	(-0.30, 0.34)	-0.16	(-0.63, 0.304)
3rd	0.22	(-0.12, 0.55)	0.22	(-0.11, 0.55)	-0.32	(-0.773, 0.13)
4th	0.17	(-0.21, 0.56)	-0.16	(-0.55, 0.23)	-0.33	(-0.78, 0.12)
Logged creatinine	2.18	(1.96, 2.40)	2.23	(2.05, 2.42)	2.24	(2.06, 2.42)
BMI	-0.01	(-0.06, 0.03)	-0.01	(-0.05, 0.03)	-0.01	(-0.05, 0.03)
Age	0.01	(0.00, 0.03)	0.02	(0.00, 0.03)	0.01	(0.00, 0.02)
Chews betelnuts	-0.40	(-0.78, -0.03)	-0.46	(-0.84, -0.09)	-0.41	(-0.78, -0.04)
ETS^† ^in the home	-0.47	(-0.79, -0.16)	-0.36	(-0.69,-0.03)	-0.43	(-0.75, -0.10)
Education^††^	-0.72	(-1.10, -0.34)	-0.70	(-1.08, -0.32)	-0.72	(-1.11, -0.33)
*GSTM1*	-0.02	(-0.37, 0.33)	0.06	(-0.30, 0.41)	-0.01	(-0.36, 0.34)
*hOGG1*	-0.03	(-0.34, 0.28)	-0.01	(-0.32, 0.29)	-0.06	(-0.37, 0.24)
*APE1*	-0.28	(-0.60, 0.04)	-0.28	(-0.59, 0.03)	-0.40	(-0.73, -0.07)

*Main effects are from three random effects Tobit models.

^†^ETS is environmental tobacco smoke.

^††^Education compared secondary or higher to primary or less

Arsenic variables were log-transformed and treated as continuous variables and also categorized into quartiles to evaluate non-linear associations. In models adjusting for potential confounders, no associations between 8-OHdG and any of the arsenic variables were observed regardless of how the variables were parameterized (Table 3). A consistent negative main effect for *APE1 *genotype was observed across water, toenail and urinary arsenic models, although it was only statistically significant for toenail arsenic (Table 3). For example, in the model evaluating toenail arsenic concentration, *APE1 148 glu/glu + asp/glu *genotype was associated with a decrease in logged 8-OHdG of 0.40 (95%CI -0.73, -0.07) compared to *APE1 148 asp/asp*. Creatinine, betel nut chewing, presence of environmental tobacco smoke in the home, and education were also predictive of 8-OHdG levels.

Interactions between genotypes and 8-OHdG were assessed and an interaction observed for *GSTM1*, but not for *APE1 *or *hOGG1*. TUA was positively associated with 8-OHdG in women with the *GSTM1 *null genotype, but not in women with *GSTM1 *positive (Table 4). Among women with *GSTM1 *null, a comparison of the second, third, and fourth quartiles of TUA to the first quartile resulted in a 0.84 increase (95% CI 0.27, 1.42), a 0.98 increase (95% CI 0.33, 1.66) and a 0.85 increase (95% CI 0.27, 1.44) in logged 8-OHdG, respectively. These results suggest a plateau effect for arsenic on 8-OHdG. A likelihood ratio test evaluating the significance of the *GSTM1 *interaction terms as a group yielded a p-value of 0.007. A model in which TUA was dichotomized at the median yielded similar results (data not shown). The same interaction was not evident in models which used toenail or drinking water arsenic instead of TUA.

**Table 4:** The effect of quartiles of arsenic on logged urinary 8-OHdG (μg/l) by *GSTM1 *genotype (N = 290)

	**GSTM1 positive**		**GSTM1 null**		**Wald p-value**^†^	**LRT p-value**^††^
		
Model*	**β**	**95% CI**	**β**	**95% CI**		
Total urinary arsenic quartile (μg/l)						0.007
1.35 – 13.3	reference		reference			
>13.3 – 22.6	-0.12	(-0.46, 0.22)	0.84	(0.27, 1.42)	0.004	
>22.6 – 47.9	-0.06	(-0.43, 0.31)	0.98	(0.33, 1.66)	0.005	
>47.9 – 431.3	-0.15	(-0.60, 0.30)	0.85	(0.27, 1.44)	0.004	
Drinking water arsenic quartile (μg/l)						0.69
0.5	reference		reference			
>0.50 – 1.5	-0.05	(-0.42, 0.31)	0.26	(-0.41, 0.93)	0.43	
>1.5 – 81.7	0.29	(-0.11, 0.69)	0.15	(-0.46, 0.75)	0.69	
>81.7 – 591	-0.21	(-0.66, 0.24)	0.002	(-0.74, 0.75)	0.63	
Toenail arsenic quartile (μg/g)						0.69
0.2 – 0.6	reference		reference			
>0.6 – 0.8	-0.19	(-0.71, 0.33)	-0.09	(-0.93, 0.75)	0.83	
>0.8 – 1.6	-0.36	(-0.87, 0.14)	-0.22	(-1.06, 0.63)	0.77	
>1.6 – 10.2	-0.49	(-1.00, 0.03)	0.05	(-0.76, 0.87)	0.26	

*Main effects are from three random effects Tobit models adjusted for logged creatinine, BMI, age, betelnut chewing, environmental tobacco smoke, and education (secondary or higher vs primary or less), and *hOGG1 *and *APE1 *genotypes.

^† ^significance test for individual interaction terms.

^†† ^significance test for the entire group of arsenic quartile by gene interactions

## Discussion

This study observed a positive association between total urinary arsenic and 8-OHdG, a biomarker of oxidative stress, among healthy women possessing the *GSTM1 *null genotype but not among women with the *GSTM1 *positive genotype. In addition, a negative main effect for *APE1 *genotype was observed. The *APE1 *variant allele was associated with a decrease in 8-OHdG which is consistent with observations that polymorphisms in the *APE1 *gene confer a decreased ability to repair oxidative damage [17].

Several epidemiologic studies have demonstrated an association between arsenic exposure and 8-OHdG [10,11]. Arsenic-related skin tumors taken from individuals currently living in arsenic-endemic areas also showed differences in 8-OHdG concentration compared to tumors from unexposed individuals [12]. Similarly, exposure of human keratinocytes to arsenite caused formation of superoxide anion, hydrogen peroxide, and 8-OHdG in a concentration- and time-dependent manner [31].

The positive association between arsenic exposure and 8-OHdG in *GSTM1 *null individuals provides evidence that there may be genetically susceptible subpopulations. *GSTM1 *mediates the generation of oxidative stress [32], can scavenge free radicals [18], and can also affect arsenic metabolism [22]. Women with the null genotype excreted a significantly higher proportion of arsenic as MMA than women with the positive genotype [33]. Increased percentage of MMA has been associated with increased risk of several arsenic-related diseases [34]. Therefore, lack of a functional *GSTM1 *enzyme may alter arsenic metabolism in favor of production of 8-OHdG.

Further evidence exists for interactions involving *GSTM1 *and oxidative stress for other environmental exposures. Bikers exposed to high ozone levels and smokers have shown positive associations with 8-OHdG DNA adduct levels only among individuals with the *GSTM1 *null genotype [35,36]. Individuals with *GSTM1 *null genotype also have an increased cancer risk for skin type I and basal cell carcinoma [19] and for smoking and lung cancer [20].

In this study, a positive association was observed only when TUA was used as the exposure metric and not for drinking water arsenic and toenail arsenic. This discrepancy may reflect differences in exposure misclassification. Toenail arsenic reflects an accumulation of historical arsenic exposure over relatively long periods of time [23]. Thus, arsenic deposited in the toenail 3–9 months prior to urinary 8-OHdG collection may not reflect the exposure relevant for urinary 8-OHdG excretion. Similarly, drinking water arsenic measurements represent a single point source of arsenic use but may not reflect total arsenic dose in the body from all routes of exposure. However, TUA measured in the same urine sample as 8-OHdG reflects recent arsenic exposure at a point in time much more relevant to the generation of the measured 8-OHdG, making it a more appropriate biomarker of exposure.

There did not appear to be a linear dose response relationship between 8-OHdG and TUA. Instead, 8-OHdG seemed to plateau at relatively low levels of urinary arsenic. However, this is a highly exposed population and it is possible that the range of arsenic exposures was insufficient to capture a linear dose response between urinary arsenic and urinary 8-OHdG before repair pathways became saturated. It is also possible that the high arsenic exposure led to an up-regulation in oxidative stress repair. Either explanation would account for the observed plateau in urinary 8-OHdG. Furthermore, it is also plausible that in the absence of *GSTM1 *enzyme alternate pathways repair oxidative damage generated from arsenic exposure.

Several limitations to the present study are worth mentioning. The sample size was relatively small, and yet the study was sufficiently powered to detect an interaction between 8-OHdG level and *GSTM1 *genotype. The failure to detect an association among women with *GSTM1 *positive genotype was not due to insufficient statistical power since this group of women was the larger stratum. Rather, it suggested that exposure to arsenic was required to reveal a different oxidative stress response between women who had the *GSTM1 *positive genotype that codes for the production of glutathione s-transferase mu and women who had the *GSTM1 *null genotype and cannot produce glutathione s-transferase mu.

Also, urinary 8-OHdG reflects oxidative DNA damage that occurs throughout the body that is corrected by DNA repair mechanisms before being excreted in urine. Thus, the effects observed in this study may reflect changes in generation of oxidative damage or changes in repair of oxidative damage or some combination of both. Furthermore, urinary 8-OHdG is a stable biomarker that is easy to measure [37] but it is not a very specific biomarker. 8-OHdG exhibits a large degree of intra-individual variability indicating a large endogenous variation of oxidative DNA damage [37,38]. In our study, the average %CV was 55.1% with a range from 4.0 to 152.8%, which was consistent with previously published work [37,38]. By using three urine samples collected on consecutive days for each woman, we were able to reduce individual variability and estimate a more precise value of an individual's base level of 8-OHdG.

In all models, creatinine was significantly associated with 8-OHdG. Creatinine is often used to adjust urinary markers for variations in urine volume or dilution by dividing the biomarker value by the creatinine value [37]. Instead, we adjusted for creatinine by including it as a log-transformed independent predictor in accordance with recent studies suggesting this is a better modeling technique, particularly when evaluating chemicals involved in one-carbon metabolism [39].

We also cannot rule out the possibility of residual confounding by sun exposure. UV exposure is a potent oxidant that can generate DNA damage that needs to be repaired. It has been implicated as a co-carcinogen with arsenic on the development of skin lesions [40]. We attempted to control for measures of sun exposure by evaluating two variables in our dataset: childhood tan level and childhood skin reaction to two hours of sun exposure. Neither of these variables appreciably changed our results; however, these variables provided only crude measurements of skin type.

## Conclusion

We observed a novel interaction between arsenic and *GSTM1 *genotype in association with urinary 8-OHdG in a population of healthy women. Urinary 8-OHdG concentration increased in response to increasing quartiles of total urinary arsenic only among women with the *GSTM1 *null genotype. These results suggest individuals that lack a functional *GSTM1 *enzyme can not detoxify arsenic well, resulting in an increase in oxidative stress. In addition, *APE1 *148Glu allele was associated with a decrease in 8-OHdG, suggesting the polymorphism affects repair of this adduct. These results contribute to a growing body of evidence that arsenic is associated with oxidative stress and repair of oxidative damage. The data also suggest it is increasingly important to consider genetically susceptible populations, as variation in genetic pathways involved in oxidative stress and arsenic toxicity may explain some of the heterogeneity in research results across studies.

## Abbreviations

8-OHdG: Urinary 8-hydroxy-2'-deoxyguanosine;

ROS: reactive oxygen species; 

DMA V: Dimethylarsinic acid;

*GSTs: *Glutathione-s transferase; 

*hOGG1: *Human 8-oxoguanine glycosylase;

*APE1*: Apurinic/apyrimidinic endonuclease;

TUA: Total urinary arsenic; 

DCH: Dhaka Community Hospital;

HPLC-HGAAS: High performance liquid chromatography-atomic absorption spectrophotometry;

As V: Arsenate; 

As III: Arsenite; 

MMA: Monomethylarsonic acid;

DMA: Dimethylarsenic acid;

ICP-MS: Inductively-coupled plasma mass spectrometer.

## Competing interests

Dr. Christiani was a paid scientific advisor for Gentra Systems. The authors have no association with Genox or Environmental Laboratory Services other than as a purchaser of services for analysis of samples.

## Authors' contributions

CB performed statistical analyses and drafted the paper. MK participated in arsenic measurement, sample quality control, and manuscript revision. PC and EH provided statistical and analytic help and guidance. QQ and MR participated in the study design and coordination of remote field operations in Bangladesh. GM organized data collection procedures, managed daily operations of field team, and processed biological samples. DC conceived of the study, participated in its design and coordination, and helped to draft the manuscript. All authors read and approved the final manuscript.
